# Enzymes, Biocatalysis and Chemical Biology

**DOI:** 10.3390/molecules25102354

**Published:** 2020-05-18

**Authors:** Stefano Serra

**Affiliations:** Consiglio Nazionale delle Ricerche (C.N.R.), Istituto di Scienze e Tecnologie Chimiche, Via Mancinelli 7, 20131 Milano, Italy; stefano.serra@cnr.it or stefano.serra@polimi.it ; Tel.: +39-02-2399-3076

Chemical transformations that take advantage of biocatalysis are of great interest to chemists. The specific activity and selectivity of the enzymes allow them to perform different chemical reactions with high regio- and stereoselectivity, and a large number of biocatalyzed industrial processes have been already established.

At the same time, we can observe the emergence of chemical biology, namely the scientific discipline spanning the fields of chemistry and biology and dealing with chemistry applied to biology.

The aim of this Special Issue is to collect studies focused on biocatalysis applied to organic synthesis, as well as research related to chemical biology. The obtained contributions dealing with biotransformations, enzymology, the stereoselective synthesis of bioactive chemical compounds, active pharmaceutical ingredients, natural products and flavours have been collected in the present Issue. 

Overall, the Issue has gathered ten research articles. 

Two of these papers deal with fungal metabolism and fungi-mediated biotransformations. A first paper from Liu et al. [1] investigates the mRNA and protein expression levels and the activities of γ-glutamyl transpeptidase and L-cysteine sulfoxide lyase in correlation with the endogenous formaldehyde content in the edible mushroom *Lentinula edodes* at different growth stages. Formaldehyde is classified as a human carcinogen and can be found in natural and processed foods. Therefore, this research provided a molecular basis for understanding and controlling the endogenous formaldehyde formation in shiitake mushroom.

A second paper from Serra and De Simeis [2] describes a study on the biotransformation of seven natural occurring apocarotenoids by means of eleven selected fungal species. The substrates, namely ionone (α-, β- and γ-isomers), 3,4-dehydroionone, damascone (α- and β-isomers) and theaspirane are relevant flavour and fragrances components. The observed transformations are mainly oxidation reactions that afford oxygenated products such as hydroxy- keto- or epoxy-derivatives. A very significant feature of the study concerns the prospective applicability of the fungi-mediated biotransformation of apocarotenoids for the synthesis of high value natural flavours. Since some ionone, damascone and theaspirane isomers are available in natural form and the biotransformation of a natural precursor is considered a ”natural method” of synthesis, the flavours obtained by means of the fungi-mediated reactions possess natural status and could be commercialized accordingly.

Four relevant works exploit the potential of some specific enzymes in biocatalyzed reactions. A first contribution from Wang et al. [3] reports on the glucosylation of ganoderic acid A, a bioactive triterpenoid isolated from the medicinal fungus *Ganoderma lucidum*.

A new ganoderic acid A-26-O-β-glucoside was produced from the O-glucosylation with recombinant BsGT110, a glycosyltransferase isolated from *Bacillus subtilis* ATCC 6633. BsGT110 was the first glycosyltransferases identified as catalyzing the glycosylation of triterpenoid at the C-26 position. Since triterpenoid glycosides may improve the bioactivity of the triterpenoid aglycone, this study could be regarded as a new tool in natural product synthesis. 

The reduction of conjugated double bonds of citral using old yellow enzyme (OYE)-mediated biotransformation was studied by Ying et al. [4]. These researchers established that a significant increase of (*R*)-enantioselectivity in the (*E*/*Z*)-citral reduction was achieved by saturation mutagenesis of P76 and R330 in OYE2y. Remarkably, the variants P76M/R330H, P76G/R330H and P76S/R330H exhibited full (*R*)-enantioselectivity in the reduction of (*E*)-citral or (*E*/*Z*)-citral.

Castro et al. [5] exploit the activity of the enzyme laccase from *Trametes versicolor* to synthetize 2,6-dimethoxy-4-(phenylimino)cyclohexa-2,5-dienone derivatives. Ten products with different substitutions in the aromatic ring were synthetized and characterized. The 3,5-dichlorinated compound showed the highest antifungal activity against the phytopathogen *Botrytis cinerea*, while the *p*-methoxylated compound had the lowest activity. In addition, the results of this research suggested that the synthesized compounds produced damage in the fungal cell wall.

Ying et al. [6] selected a reductase for the synthesis of a specific chemical intermediate. The recombinant carbonyl reductase from *Rhodococcus erythropolis* WZ010 (ReCR) demonstrated strict (*S*)-stereoselectivity. The enzyme catalyzed the irreversible reduction of N-Boc-3-piperidone to (*S*)-N-Boc-3-hydroxypiperidine, which is the key chiral intermediate in the synthesis of ibrutinib, an active pharmaceutical ingredient. This NAD(H)-specific enzyme proved to be active within broad ranges of pH and temperature and had remarkable activity in the presence of higher concentration of organic solvents. 

A further four works deal with the characterization of specific enzymes and with the study of their activity. Hu et al. [7] described the role of polyphenol oxidase in the browning reaction of orange juice. The research is also finalized to determine the methods of inactivation of the same enzymes. Fluorescence spectroscopy, circular dichroism and dynamic light scattering were used to investigate the ultrasonic effect on polyphenol oxidase activity, demonstrating that this treatment causes inactivation of the enzyme.

The correlation between activity and enzyme modification was investigated by Sun et Al. [8]. The sweet potato β-amylase was modified by six types of methoxy polyethylene glycol to enhance its specific activity and thermal stability. The aims of the study were to select the optimum modifier, optimize the modification parameters and further investigate the characterization of the modified sweet potato β-amylase. The results showed that methoxy polyethylene glycol maleimide (molecular weight 5000, Mal-mPEG5000) was the optimum modifier.

Krikštaponis et al. [9] reported a study on the 7-hydroxycoumarin catabolic pathway in *Pseudomonas* sp. 7HK4 bacteria. New metabolites and genes responsible for the degradation of 3-(2,4-dihydroxyphenyl)-propionic acid have been isolated and identified. The results show that the degradation of 7-hydroxycoumarin in *Pseudomonas* sp. 7HK4 involves a distinct metabolic pathway, compared to the previously characterized coumarin catabolic routes through a unique flavin-binding ipso-hydroxylase. Thus, the study provides new insights into the degradation of hydroxycoumarins by soil microorganisms.

Finally, He et al. [10] propose a new rapid assay to measure the activity of adenylate kinase. The latter enzyme plays a fundamental role in cellular energy and nucleotide homeostasis. The study shows a spectrophotometric analysis technique to determine enzyme activity with bromothymol blue as a pH indicator.

Overall, these ten contributions provide the reader with relevant fresh insights on the use of enzymes and on the importance of the biocatalysis. Furthermore, studies regarding the chemical biology are well represented within this Special Issue. 
